# Three-Dimensional Multi-Doped Porous Carbon/Graphene Derived from Sewage Sludge with Template-Assisted Fe-pillared Montmorillonite for Enhanced Oxygen Reduction Reaction

**DOI:** 10.1038/s41598-017-03845-z

**Published:** 2017-06-23

**Authors:** Meiqing Chen, Pingxiao Wu, Liya Chen, Shanshan Yang, Langfeng Yu, Yuefei Ding, Nengwu Zhu, Zhenqing Shi, Zehua Liu

**Affiliations:** 1 School of Environment and Energy, South China University of Technology, Guangzhou, 510006 P.R. China; 2 The Key Lab of Pollution Control and Ecosystem Restoration in Industry Clusters, Ministry of Education, Guangzhou, 510006 P.R. China; 3 Guangdong Provincial Engineering and Technology Research Center for Environmental Risk Prevention and Emergency Disposal, South China University of Technology, Guangzhou Higher Education Mega Centre, Guangzhou, 510006 P.R. China; 4 Guangdong Environmental Protection Key Laboratory of Solid Waste Treatment and Recycling, Guangzhou, 510006 China; 5 Guangdong Engineering and Technology Research Center for Environmental Nanomaterials, Guangzhou, 510006 China

## Abstract

Three-dimensional multi-doped porous carbon/graphene (Fe-Mt-SS-C) was prepared by carbonization of sewage sludge with template-assisted Fe-pillared montmorillonite. The material consisted of nanosheet- and particle- carbon had a high specific surface area (784.46 m^2^·g^−1^) and hierarchical porous structure of micro-, meso- and macropores. The prepared Fe-Mt-SS-C had a high degree of graphitization and large amount of defect atoms. The pyrolysis process made full use of the C, N, Fe, and S by turning them into the carbon framework of the as-obtained material *in situ*. Template-assisted Fe-pillared montmorillonite contributed to more characteristics of morphology and composition on Fe-Mt-SS-C than other three materials (SS-C, Mt-SS-C and Fe-SS-C), and enhanced the electrocatalytic ORR activity by providing more adsorption sites and the electronic structure, resulting in the increase of conductivity and electrochemical activity. The ORR activity performance of Fe-Mt-SS-C, including the value of onset potential (0.03 V) and E_1/2_ (−0.09 V), was better than that of commercial 20 wt% Pt/C (−0.02 V and −0.18 V, respectively). Moreover, the Fe-Mt-SS-C possessed excellent durability and outstanding immunity toward methanol crossover effects. Therefore, the resultant Fe-Mt-SS-C has great potential to applied as a high-efficiency ORR electrocatalyst, more importantly, it realizes the utilization of the sludge at the same time.

## Introduction

With the continuous development of society, energy depletion and environmental pollution get worse. It has no doubt that increasing demands for clean energy^1^. The fuel cell with significant advantage stands out^2^. Alkaline fuel cell is a kind of highly potential energy converter with low emission and high efficiency. But cathode catalytic materials of alkaline fuel cell, such as Pt/C catalyst, are expensive and its catalytic activity can’t meet the actual demands, which are the main limiting factors in commercialization of alkaline fuel cell^3–5^. Therefore, the preparing low cost, excellent activity and high methanol cathode catalytic materials for ORR are the keys to solve the existing problems and achieve the commercialization of fuel cell.

Carbon materials are widely used as cathode catalytic materials in the fuel cell because of their numerous sources, low cost and good stability^6, 7^. A large number of studies have shown that modification by doping with metal or nonmetal heteroatoms on carbon materials can achieve optimization of surface performance, so that getting the catalyst with high catalytic activity applies to oxygen reduction reaction^8^. For example, Carbon-based nanomaterials doped metals heteroatoms (Fe, Mn and Co)^9, 10^ and nonmetal heteroatoms (N, S, P, etc.)^11–16^ have been observed as promising electrocatalysts for ORR on account of their superior electrocatalytic performance, extraordinary methanol tolerance, remarkable long-term stability, and importantly low cost. Furthermore, many excellent reviews have demonstrated that multi-doped carbon-based nanomaterials (Fe-N-C, S-N-C, P-N-C, etc.)^17–21^ can markedly enhance the ORR activity of carbon-based materials. However, most of the multi-doped carbon nanomaterials were developed by using multiple hazardous chemicals as precursors^22^. There is thereby an urgent need but it is still a significant challenge to prepare a low-cost multi-doped carbon-based nanomaterial with high ORR performance by utilizing eco-friendly raw material or waste^23–25^.

With the treatment capacity of sewage greatly increased, urban sludge problem is increasingly outstanding^26^. Traditional disposal methods of sludge (land sanitary landfill, incineration and Land utilization etc.) not only have the defects of covering a large area, high cost and security risk, but also can’t fundamentally realize the “reduction, stabilization, harmless and recycling” of sludge^27^. Resource utilization is the main trend to solve the problem of sludge. As a result, the conversion of sludge from waste pollutants into the available resources and maximize the value of available components released from the sludge are the ultimate goal of solving the municipal sludge pollution. Sewage sludge consists mainly of organic matter (up to 50–70%), protein and inorganic oxide (containing nitrogen, phosphorus and sulfur)^28, 29^. Sludge pyrolysis can convert it into heteroatoms-doped carbon material, which can be used as a fuel cell cathode catalyst^30^. There are many excellent studies have investigated the template synthesis strategy in the field of producing micro-/meso-/macroporous carbons with nanoarchitecture^31–33^. Inorganic solid materials such as zeolites and mesoporous silica have been triumphantly applied as templates to obtain nanoporous carbon materials^34^. Furthermore, montmorillonite (Mt), especially the modificated montmorillonite with expandable interlayer structure and chemical inertness siloxane surface, is thought to be the ideal template in converting carbon-rich organic compounds into three-dimensional carbon nanomaterials^35, 36^. This green technology for recycling of sewage sludge has great research significance and a broad application prospect. However, there is short of relevant research.

In this study, we demonstrated a facile method for developing multi-doped porous carbon/graphene materials for oxygen reduction reaction by using sewage sludge as the sources of carbon and doped heteroatoms, and synergistic effect of template assist from Fe-pillared montmorillonite. The mixture of Fe-pillared montmorillonite and sewage sludge was used to prepare three-dimensional carbon nanomaterial with graphene-style structure. On the one hand, it can make full use of carbon, nitrogen and iron in sludge. On the other hand, it can also take advantage of expandable interlayer structure and the template effect of Fe-pillared montmorillonite. The resultant multi-doped porous carbon/graphene materials had a high specific surface area and doped with heteroatoms exhibited excellent high ORR activity, remarkably ability for immune methanol crossover, and superior durability in alkaline medium, as compared to the other three prepared materials (SS-C, Mt-SS-C and Fe-SS-C) and the commercial 20 wt% Pt/C catalyst. Overall, this study not only puts forward a facile method to prepare multi-doped porous carbon/graphene materials by using sewage sludge as the precursor for oxygen reduction reaction, but also revealed an innovative and green way for sludge recycling use. Preliminary results have shown that the Fe-Mt-SS-C prepared using mixture with m_Fe-Mt_/m_sludge_ of 0.2 and pyrolyzed under 800 °C exhibited more effective oxygen reduction reaction removal than other materials (details are presented in Supporting Material Fig. S1). Fe-Mt-SS-C appeared below was prepared under optimum condition if there was no special instruction.

## Results

### Morphology and structure analysis

**Figure 1:**
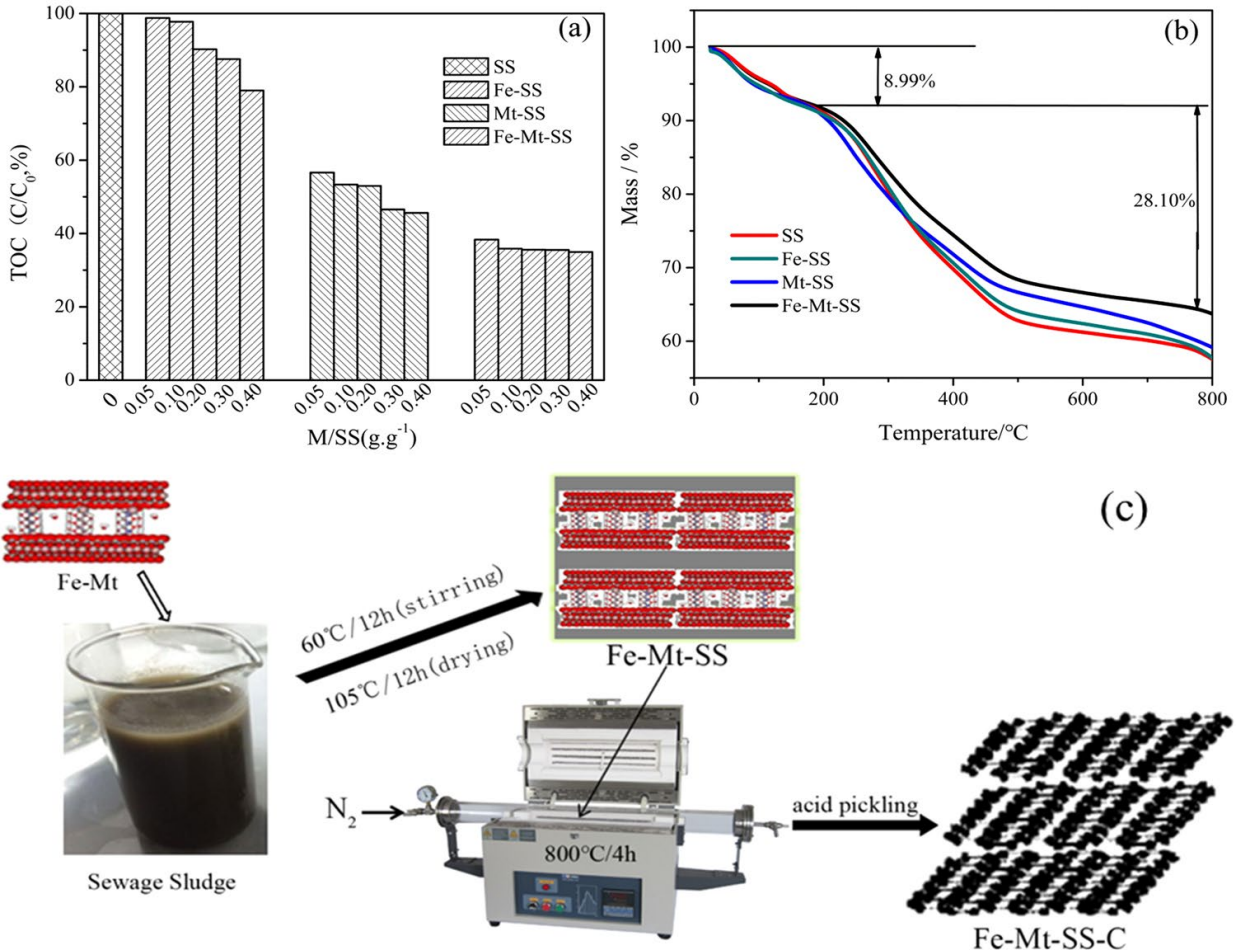
(**a**) TOC concentration of centrifugation from sewage sludge and after adding difference-proportion Mt, FeOOH and Fe-Mt, (**b**) TG curves of SS, Fe-SS, Mt-SS and Fe-Mt-SS, (**c**) schematic diagram of the preparation of the Fe-Mt-SS-C.

As Fig. 1a illustrated, TOC concentration of supernatant decreases with the increase of the additives (Mt, FeOOH or Fe-Mt)/SS ratios, indicating that Mt, FeOOH and Fe-Mt can adsorb organic compounds in sludge liquid, which has demonstrated in previously research works^37, 38^. The sequence of adsorption capacity was in the following order Fe-Mt > Mt > FeOOH in all additives/SS ratios, suggesting that Fe-Mt can reduce the risk of pollution derived from sludge centrifugal liquid, as well as make full use of carbon source for further pyrolysis.

The analyses of thermogravimetry (TG) on four pyrolysis precursors (SS, Fe-SS, Mt-SS and Fe-Mt-SS) were conducted. As shown in Fig. 1b, there are two mass loss steps: from 30 to 200 °C, the weight loss could be attributed to removal of physically adsorbed water; the gradual mass loss beginning at 200 °C resulted from removal of oxygen-containing functional groups such as OH, C-O, COOH, decomposition of various hydrated compounds and transformation of organic compounds into a carbon material^11, 39, 40^. It should be pointed out that the weight loss at each step of Fe-Mt-SS was lower than the other three materials (SS, Fe-SS, Mt-SS), implying that Fe-Mt can maximize preventing the loss of available component in the sludge. The preparation of the Fe-Mt-SS-C followed schematic diagram exhibited as Fig. 1c.

**Figure 2:**
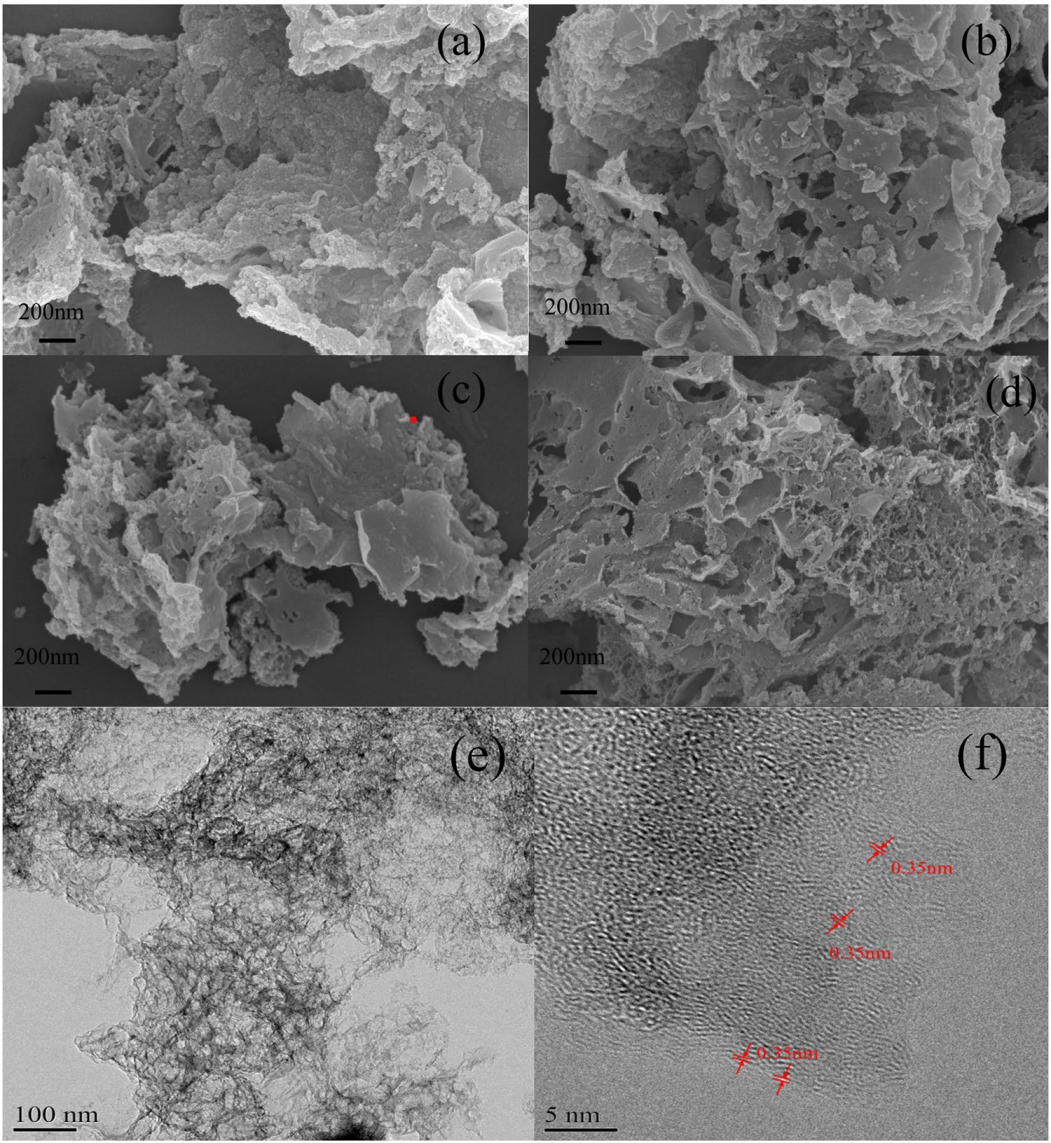
(**a**–**d**) FE-SEM images of SS-C, Fe-SS-C, Mt-SS-C and Fe-Mt-SS-C, respectively. (**e**) TEM image of Fe-Mt-SS-C. (**f**) HR-TEM image of Fe-Mt-SS-C.

As displayed in Fig. 2a–d, there is obviously difference in the morphologies of the four as-obtained materials (SS-C, Fe-SS-C, Mt-SS-C and Fe-Mt-SS-C). The SS-C mainly forms by a number of carbon particles with diameter of 50 nm and the serious phenomenon of reunion can be found, exhibiting low degree of graphitization. Fe-SS-C and Mt-SS-C primarily consist of nanosheet-carbon with a small amount of particles, indicating that FeOOH and Mt are not only beneficial to the carbonization of the organism in sludge, but also can promote degree of graphitization for carbon. It is worthwhile mentioning that Fe-Mt-SS-C has apparent three-dimensional structure and mainly included uniform graphene-like nanosheets coupled with carbon network composed of tiny particles, which may ascribe to the template assist of Fe-Mt. There were three kind of pore: the macropores generated by removing silicon dioxide in sludge at acid pickling and the stack of graphene-like nanosheets; mesopores and micropores resulted from acid pickling of nano-iron particles on Fe-Mt. A typical TEM image of Fe-Mt-SS-C, as presented in Fig. 2e, indicates overlapping of porous carbon nanosheets in uneven state. Figure 2f reveals the lattice spacing of Fe-Mt-SS-C is approximately 0.35 nm that is coincident with the reported results about the lattice spacing of graphene, which further proves the formation of graphene-like nanosheets on Fe-Mt-SS-C^41, 42^.

**Figure 3:**
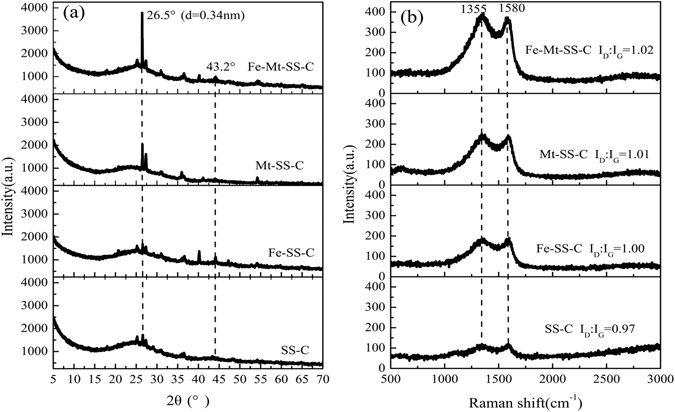
(**a**) XRD patterns and (**b**) Raman spectra of SS-C, Fe-SS-C, Mt-SS-C and Fe-Mt-SS-C, respectively.

As illustrated in Fig. 3a, the XRD patterns of four materials (SS-C, Fe-SS-C, Mt-SS-C and Fe-Mt-SS-C) exhibit a peaks at 26° labelled as (002) of graphitic-like structure (JCPDS, Card No. 41-2487), while the peak intensity at 26° suggested the degree of graphitization was in the order of Fe-Mt-SS-C > Mt-SS-C > Fe-SS-C > SS-C. Fe-Mt-SS-C appears a peak at approximately 43°, corresponding to the (100) crystal plane, illustrating that the degree in the crystal plane was ordered^43, 44^. Figure 3b shows Raman spectras of the obtained materials, there are two bands at 1355 cm^−1^ (D-band) related to sp3 defects and 1580 cm^−1^ (G-band) corresponding to the in-plane vibration of sp2 carbon. It was noteworthy that the peak intensity of G-band and the ratio of the D- and G-band intensities (I_D_/I_G_) on behalf of a quantitative assessment of the defect concentration in carbon materials is with the order of Fe-Mt-SS-C (1.02) > Mt-SS-C (1.01) > Fe-SS-C (1.00) > SS-C (0.97), indicating Fe-Mt-SS-C exists a larger degree of graphitization and a larger amount of defect atoms^45, 46^. The high degree of graphitization and defect structure were ascribed to the contribution of the template effect originated from Fe-Mt. The discoveries of XRD patterns and Raman spectras were consistent with the observation of SEM and TEM.

**Figure 4:**
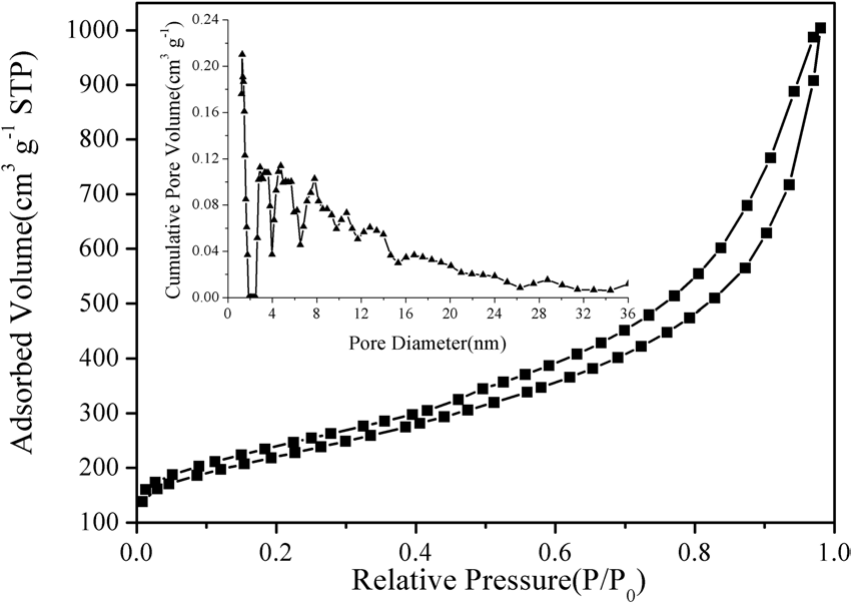
(Inset) pore size distribution and N_2_ adsorption-desorption isotherms of Fe-Mt-SS-C.

The adsorption measurements were used to further investigate the feather of the obtained carbon materials. As can be seen from Fig. 4, the N_2_ adsorption/desorption analysis isotherm of Fe-Mt-SS-C presented a type IV curve with a gradually increased of positive slope as P/P_0_ (0.43–0.98) increased, suggesting that there are good transport properties among the microspores, mesoporous and macroporous channels. The Fe-Mt-SS-C had a BET surface area of 784.46 m^2^·g^−1^, according to the Brunauer-Emmett-Teller method, which was higher than that of the other three samples with the order of Fe-Mt-SS-C > Mt-SS-C > Fe-SS-C > SS-C (Table 1). The pore-size distribution curve was illustrated as inset in Fig. 4 and the pore diameters were centered mainly at 1, 3, 5–13, 17 and 30 nm, implying a hierarchical porous structure of Fe-Mt-SS-C that will contribute well to the electrochemical performance because the micropores hold charges, mesopores quicken the kinetic process of ion diffusion, and the open channels of macropores act as ion-buffering depositories^47–49^. As Table 1 listed, the content of the elements (N, C, S and Fe) on Fe-Mt-SS-C is most compared with the other three obtained materials. Considerable research efforts have found that the introduction of heteroatoms (e.g., N, O, S, Fe, etc.), especially nitrogen, would play a role of promoting in the oxygen reduction reaction^39, 50–52^.

**Table 1:** Properties of the as-prepared materials.

samples	SS-C	Fe-SS-C	Mt-SS-C	Fe-Mt-SS-C
SBET (m^2^.g^−1^)	642.19	650.49	744.07	784.46
average pore size (nm)^a^	5.51	5.80	5.83	7.92
**conc**. (**wt%**)				
N^b^	3.25	3.77	3.07	4.48
C^b^	66.40	63.02	61.17	66.48
S^b^	4.70	3.88	3.80	5.67
H^b^	1.49	0.65	0.49	1.20
P^c^	0.57	1.10	1.96	0.14
Fe^c^	1.78	2.29	0.83	4.07
Mg^c^	0.05	0.16	0.05	0.16
Al^c^	1.12	2.04	0.99	2.10
Mn^c^	0.01	0.01	0.01	0.02
Cu^c^	1.72	1.08	0.17	1.11
Ca^c^	0.05	0.07	0.03	0.07
K^c^	0.02	0.02	0.02	0.01
Ni^c^	0.12	0.07	0.02	0.06
Cr^c^	0.14	0.19	0.37	0.23

^a^Calculated from the Barrett-Joyner-Halenda equation using the desorption isotherm. ^b^The contents of C, N, and S were obtained by Elemental Analyze. ^c^The contents of metals were obtained by ICP.

**Figure 5:**
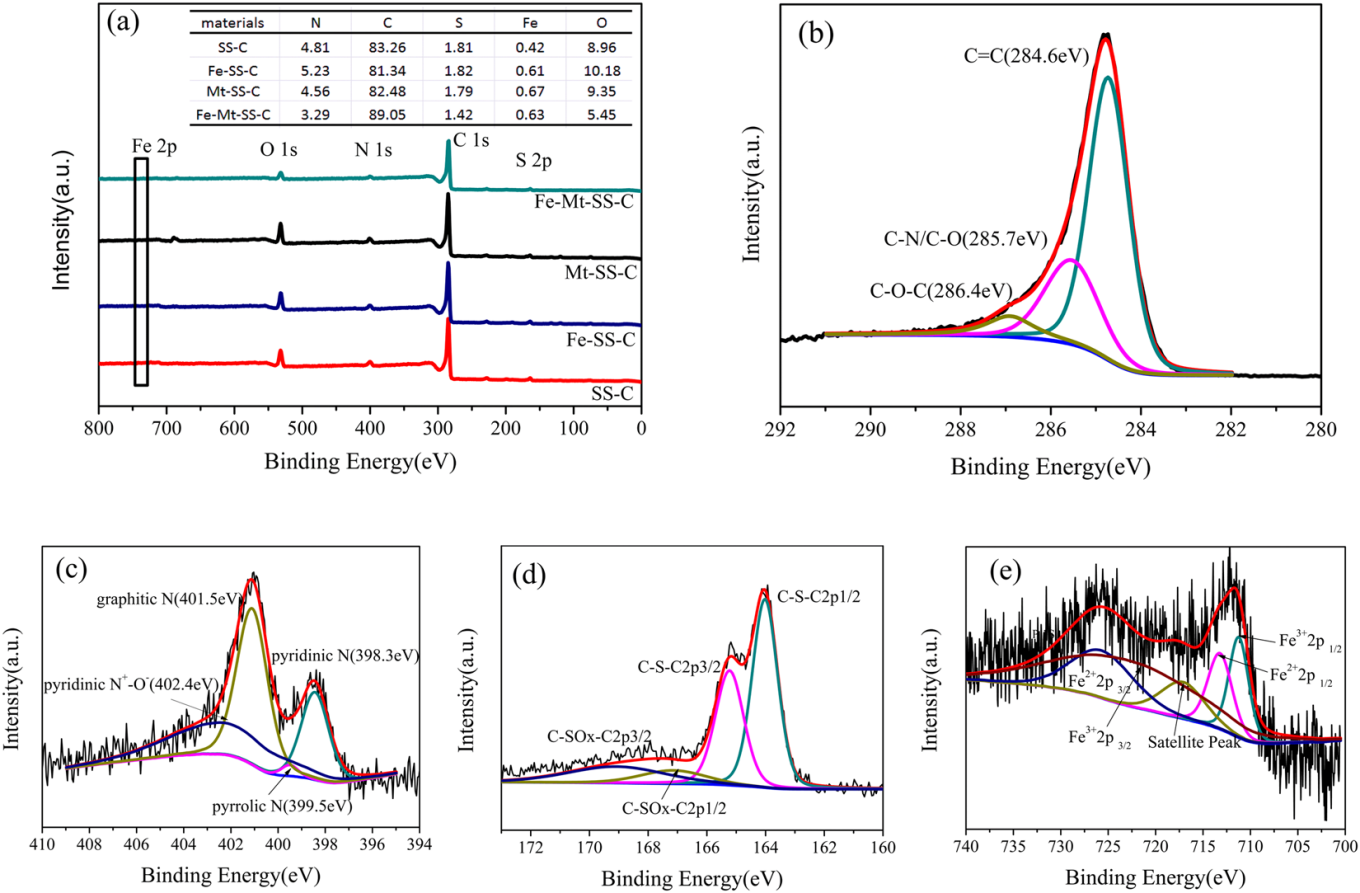
(**a**) XPS survey of SS-C, Fe-SS-C, Mt-SS-C, Fe-Mt-SS-C and (**b**–**e**) high-resolution spectra of C, N, S and Fe in Fe-Mt-SS-C.

Surface analysis of the prepared materials was detected using X-ray photoelectron spectroscopy (Fig. 5). As Fig. 5a illustrated, the four materials exhibit apparent C1s, O1s, N1s, S2p and Fe2p peaks, in which no Si2p peak is found in the XPS spectrum, indicating SiO_2_ had been pickling completely. The corresponding content was listed in the inset table. It must be mentioned that the mass percentages of N1s, S2p and Fe2p in Fe-Mt-SS-C were the least comparing with the other samples (SS-C, Fe-SS-C and Mt-SS-C), which was contrary to the results of Table 1, demonstrating that part of these elements distribute within the carbon frame, which was conducive to oxygen reduction reaction. As Fig. 5b revealed, the most of the carbon in Fe-Mt-SS-C was in the form of C=C and C-N (Table S1), suggesting that the degree of graphitization was high and this two forms of carbon would play a major role in promoting electrochemical performance^44^. As shown in Fig. 5c, the N1s spectrum exhibits four contributions, pyridinic N, pyrrolic N, graphitic N and pyridinic oxide (pyridinic N^+^-O^−^) located at 398.3 eV, 399.5 eV, 401.5 eV and 402.4 eV, respectively^12^. Notably, the content of pyridinic N and graphitic N reached up to 66.5% in Fe-Mt-SS-C (Table S2), the high percentage of pyridinic N and graphitic N was beneficial to oxygen reduction reaction. High-resolution spectra of S in Fe-Mt-SS-C was displayed as Fig. 5d, S2p1/2 and S2p3/2 located at 163.9 eV and 165.2 eV further verified that S atoms were triumphantly doped into the carbon framework^53^. Moreover, the coexistence of Fe^3+^ and Fe^2+^ is illustrated as Fig. 5e, suggesting Fe hadn’t been pickling completely that is favor of boosting the electrochemical performance^54^. Thus it can come to the conclusion that the pyrolysis process made full use of the C, N, Fe, and S by turning them into the carbon framework of Fe-Mt-SS-C, which can provide electrocatalytic active sites, such as C=C, C-N, pyridinic N, graphitic N, C-S-C, etc.

### Electrocatalytric properties analysis

**Figure 6:**
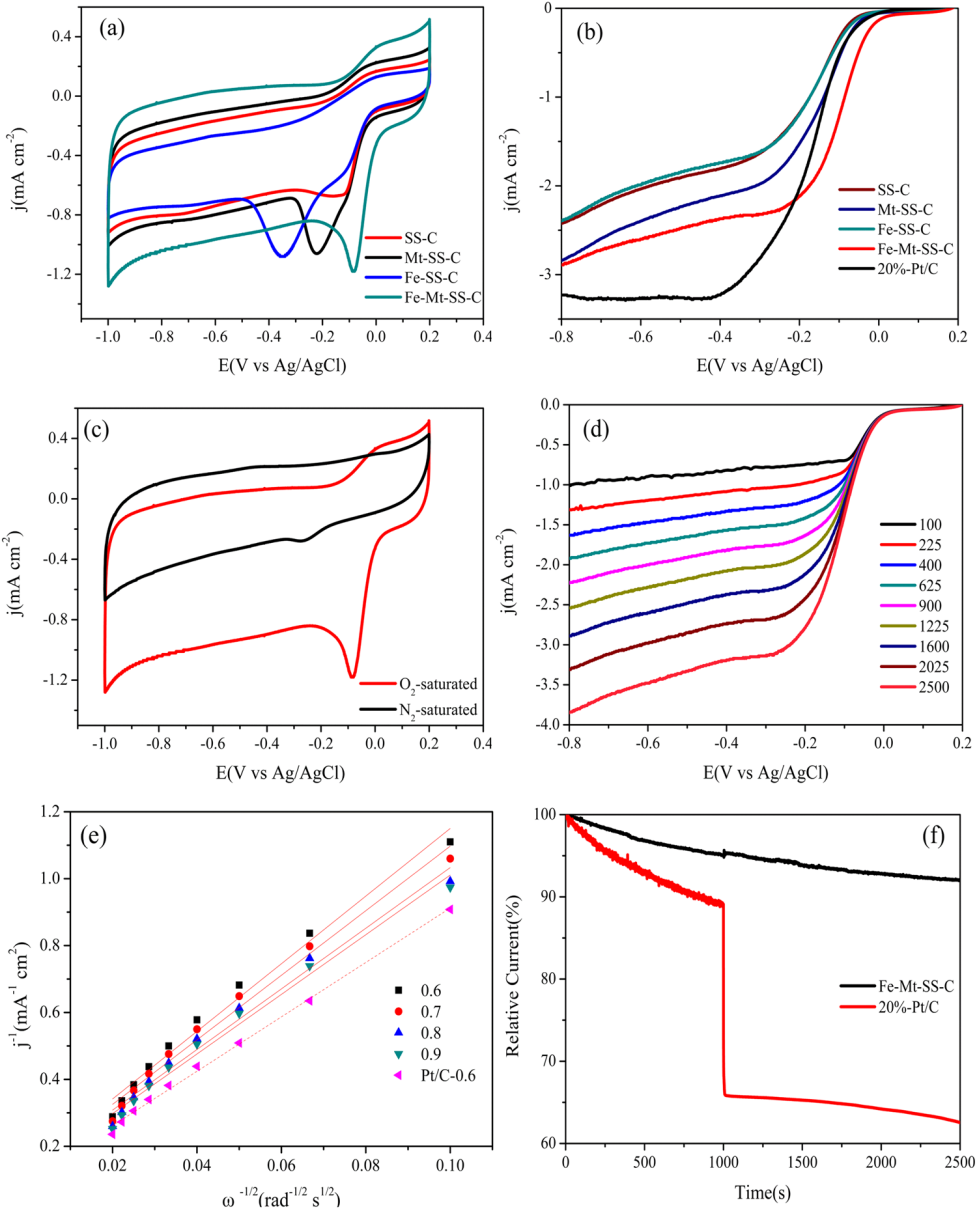
(**a**) Cyclic voltammograms of as-obtained materials in O_2_-saturated 0.1 M KOH at a scan rate of 10 mV·s^−1^; (**b**) RRDE voltammograms of as-prepared materials and Pt/C at the rotation rate of 1600 rpm. Scan rate: 10 mV·s^−1^; (**c**) Cyclic voltammograms of a Fe-Mt-SS-C in O_2_-saturated and N_2_-saturated 0.1 M KOH at a scan rate of 10 mV·s^−1^, respectively; (**d**) steady state polarization curves of O_2_ reduction for Fe-Mt-SS-C in O_2_-saturated 0.1 M KOH at different rotation rates. Ring potential was set at +0.5 V. Potential scan rate was 10 mV·s^−1^. (**e**) Koutecky-Levich plots of Fe-Mt-SS-C at different electrode potentials. (**f**) Chronoamperometric responses of Fe-Mt-SS-C and Pt/C electrodes with the addition of 3 M methanol.

The electrocatalytic ORR activity of as-obtained materials was firstly evaluated by cyclic voltammogram (CV) measurements. As depicted in Fig. 6a, Fe-Mt-SS-C illustrates larger double-layer current density than these of SS-C, Fe-SS-C and Mt-SS-C in O_2_-saturated 0.1 M KOH, ascribing to the three-dimensional multi-doped porous carbon/graphene with larger surface area as the result of the template-assisted Fe-pillared montmorillonite. Comparative studies of the ORR of as-prepared materials with Pt/C were carried out at the rotation rate of 1600 rpm in O_2_-saturated 0.1 M KOH. As exhibited in Fig. 6b, the onset potential is 0.03 V for Fe-Mt-SS-C, −0.02 V for Mt-SS-C and Pt/C, and −0.03 V for Fe-SS-C and SS-C, respectively. And the limiting diffusion current density at −0.4 V increased in the order of 1.76 mA·cm^−2^ (SS-C) < 1.81 mA·cm^−2^ (Fe-SS-C) < 2.11 mA·cm^−2^ (Mt-SS-C) < 2.38 mA·cm^−2^ (Fe-Mt-SS-C) < 3.25 mA·cm^−2^ (Pt/C). It should be pointed out that the value of the half-wave potential (E_1/2_) of Fe-Mt-SS-C was −0.09 V (vs Ag/AgCl), whereas that of 20 wt% Pt/C was −0.18 V. It can come to a conclusion that the ORR activity performance of Fe-Mt-SS-C, including the value of onset potential and E_1/2_, was better than that of SS-C, Fe-SS-C, Mt-SS-C and commercial 20 wt% Pt/C.

The ORR activity of Fe-Mt-SS-C was estimated by using CV in O_2_- and N_2_-saturated 0.1 M KOH. As shown in Fig. 6c, the Fe-Mt-SS-C displays an oxygen reduction peak at −0.08 V in saturated with O_2_, while in N_2_-saturated 0.1 M KOH, the Fe-Mt-SS-C shows a featureless voltammograms current within the potentials of −1.0 V to 0.2 V (vs Ag/AgCl), which significantly illustrates the ORR activity was effective on Fe-Mt-SS-C electrode in alkaline medium with O_2_ ambience. To acquire in-depth knowledge of the electrocatalytic activity on Fe-Mt-SS-C, linear sweep voltammetry (LSV) tests were conducted at given speeds from 100 rpm to 2500 rpm in O_2_-saturated 0.1 M KOH electrolyte. As presented in Fig. 6d, a typical increasing current density with higher rotation speed is displayed, which can be explained by the enhanced oxygen transport at the electrode surface as shortened diffusion distance at high speeds. The result indicated that the ORR of the Fe-Mt-SS-C is diffusion controlled process. The corresponding K-L plots (J^−1^ vs. ω^−1/2^) obtained from the LSV curves of the Fe-Mt-SS-C exhibited good parallel straight lines (Fig. 6e), indicating a first-order reaction kinetics with the concentration of dissolved oxygen and the same number of transferring electron during ORR at different electrode potentials. On the basis of the slopes gained from the K-L equations, the number of electron transferred was calculated to be about 3.6, reflecting that the ORR was the four-electron route, which was comparable to 20wt% Pt/C catalyst^55^.

The tolerance against methanol crossover of Fe-Mt-SS-C and Pt/C catalysts towards the ORR was investigated by chronoamperometric measurements. As illustrated in Fig. 6f, the methanol crossover of the Fe-Mt-SS-C is significantly different from that of the Pt/C catalyst, which is subjected to a sharp 25% decrease in current when the addition of 3 M methanol into the electrolyte. Notably, the catalytic current density of the Fe-Mt-SS-C exhibits a good tolerance. These findings suggest that the Fe-Mt-SS-C possesses excellent durability and immunity toward methanol effects, thus having potential to apply as a high-efficiency ORR electrocatalyst.

## Discussions

The excellent ORR activity of the Fe-Mt-SS-C can be ascribed to the followed three factors. Firstly, the ORR performance of Fe-Mt-SS-C ascribed to high conductivity and electrochemical activity resulted from the 3D structure formed by the nanosheet carbon, the high specific surface area and the porous nature (macrospores, mesopores and microspores). Secondly, according to TEM and Raman results, Fe-Mt-SS-C had a high degree of graphitization and large amount of defect atoms assisted the template of Fe-Mt, which served as adsorption sites in ORR^56, 57^. Thirdly, based on the findings of XPS and EA, the C, N, Fe and S had doped into the carbon framework of the as-obtained Fe-Mt-SS-C, which is advantageous to the electronic transmission within the material. The resultant charge separation promotes the adsorption of O_2_ over the catalyst and hence improves the ORR property. It had highest total-nitrogen amount and maximal content of pyridinic N and graphitic N that were in favor of oxygen reduction reaction. The coexistence of S and Fe atoms in the carbon framework affected the electronic structure of carbon materials, resulting in the increase of conductivity and electrochemical activity^58^. Thus it can come to the conclusion that the pyrolysis process take full advantage of the C, N, Fe and S in the sewage sludge by doping them in the carbon framework of the as-obtained material, which can provide electrocatalytic active sites, such as C=C, C-N, pyridinic N, graphitic N, C-S-C, C-Fe, etc.^59, 60^. Therefore, the catalytic activity of Fe-Mt-SS-C is much better than that of SS-C, Mt-SS-C, Fe-SS-C. In conclusion, the excellent ORR activity of Fe-Mt-SS-C was due to the synergistic effect of three-dimensional porous structure, a high degree of graphitization with large amount of defect atoms and multi-doped (N, S, and Fe, etc.).

Three-dimensional multi-doped porous carbon containing activated nanoparticles and graphene-like nanosheets were acquired by template-assisted Fe-pillared montmorillonite for sewage sludge via calcination methods. The template-assisted Fe-pillared montmorillonite made a greater contribution to the structure with unique characteristic and the C, N, Fe, and S doping into the carbon framework of Fe-Mt-SS-C than the other three prepared materials (SS-C, Mt-SS-C and Fe-SS-C), and enhance the ORR activity of Fe-Mt-SS-C by providing higher specific surface area and offering more active sites. The value of onset potential (0.03 V) and E_1/2_ (−0.09 V) of Fe-Mt-SS-C was better than that of commercial 20 wt% Pt/C (−0.02 V and −0.18 V, respectively). The Fe-Mt-SS-C also exhibited outstanding durability and excellent methanol tolerance compared to Pt/C. As a result, the Fe-Mt-SS-C prepared by low-cost method with superior electrochemical performance reveals that the obtained material is good candidate for oxygen reduction reaction, this work may also provide a fundamental and facile method on resource utilization of sludge.

## Methods

### Materials

Mt used in this study was obtained from Nanhai, Guangdong province, China. Its cation exchange capacity (CEC) and specific surface area is 0.78 meq·g^−1^ and 71 m^2^·g^−1^, respectively. The chemical composition of Mt is 50.8%, 32.4%, 6.75%, 1.86%, 1.7%, 0.09%, 0.72% and 2.07% for O, Si, Al, Mg, Ca, K, Na and Fe, respectively, which was determinated by X-ray fluorescence. Sewage sludge sample was supplied by the Lijiao wastewater treatment, located in Guangzhou, China. Fe(NO_3_)_3_, Na_2_CO_3_, HF (40 wt%) and HCl (37 wt%) were purchased from Guangzhou Chemical Reagent Factory (Guangzhou, P. R. China). All the chemicals were of analytical grade and were used without further purification.

### Preparation of Fe-pillared montmorillonite

Fe-pillared montmorillonite was prepared according to our previous report^17^. 1 mol L^−1^ Na_2_CO_3_ solution was added dropwise into 1 mol L^−1^ Fe(NO_3_)_3_ solution under magnetic stirring, thermostated at about 65 °C for 24 h. The molar ratio of [OH^−^]/[Fe^3+^] was 2.0, the hydroxyl solution was slowly added to 2 wt% montmorillonite suspension at a [Fe]/clay ratio of 10 mmol kg^−1^, which was prepared in advance in an ultrasonic bath for 30 min, under vigorously stirring. The obtained Fe-pillared montmorillonite suspension was stirred for another 2 h and aged for 24 h at 65 °C. The obtained samples were dried at 65 °C overnight, ground to 200-mesh, labeled as Fe-Mt.

### Preparation of multi-doped porous carbon/graphene materials

100 ml of sewage sludge suspension with 5%-solid-state rate was mixed with 2 wt% Fe-Mt suspension at a m_Fe-Mt_/m_sludge_ of 0.2. The obtained suspension was stirred at 60 °C for 12 h followed centrifugal, and the supernatant was sampled for TOC analysis. The obtained solid mixture was dried at 105 °C for12 h, ground to 200-mesh, noted as Fe-Mt-SS. For comparison, sewage sludge, Mt mixed sludge at a m_Mt_/m_sludge_ of 0.2 (Mt-SS) and iron hydroxyl solution mixed sludge at a m_Fe_/m_sludge_ of 0.2 (Fe-SS) were dealt with the same process. The obtained precursors (SS, Mt-SS, Fe-SS and Fe-Mt-SS) were pyrolyzed in a quartz boat at 800 °C for 4 h at a rate of 5 °C·min^−1^ under N_2_ flow and cooled down to room temperature. In order to liberate carbon materials, the pyrolysis products were washed by 20 wt% HF followed by 18 wt% HCl for 4 h repeat three times, then filtration and drying. The resulting carbon/graphene materials were donated as SS-C, Mt-SS-C, Fe-SS-C and Fe-Mt-SS-C, respectively.

### Characterizations

Thermo-gravimetric and differential scanning calorimetry (TG-DSC) analyses were carried out by using a thermal analysis meter (STA-449F3, Netasch, Germany) under N_2_ atmosphere with a heating rate of 5 °C·min^−1^ from 30 to 1000 °C. A field emission scanning electron microscopy (SEM, Carl Zeiss, Germany) with an accelerating voltage of 20 kV combined and a transmission electron microscopy (TEM, JEM-3010, JEOL Ltd., Japan) with an accelerating voltage of 200 kV were used to investigate the surface morphology of the materials. The crystalline structure of the products were taken on a D8-Advance X-ray diffractometer, operated on Cu Ka radiation (λ = 1.5406 Å) at 40 kV and 40 mA, with a scan step of 0.02°, a rate of 19.2 s per step and a scan range from 5° to 70°. A FTIR spectrometer (American 96 Thermo-electron Corporation) was used to analyze Fourier-transform infrared (FTIR) spectroscopy, over the spectral range of 4000–400 cm^−1^. Raman spectras were obtained on a Renishaw Micro-Raman spectroscopy (Renishaw in Via Reflex) using 514 nm laser excitation. Nitrogen adsorption-desorption experiments for surface and porosity quantification were performed with Chemisorption Surface Area Analyzer (ASAP 2010, Micromeritics, USA). The surface areas were estimated by using the Brunauer-EmmettTeller method. The X-ray photoelectron-spectras (XPS) analysis were identified by an X-ray photoelectron spectrometer (AES430S, ANELVA, Japan).

### Electrochemical measurements

Cyclic voltammetry (CV) and linear sweep voltammetry (LSV) measurements were handled in a three-electrode system on an electrochemical workstation (CHI 660E, Shanghai Chenhua Instrument Co, China) with a rotating disk electrodeimum wire. A platinum foil and Ag/AgCl were used as the counter electrode and reference electrode, respectively. The working electrode was prepared as following: The materials (2 mg) were firstly ultrasonically dispersed in ethanol (1 mL) to obtained homodispersed dispersion and then Nafion emulsion (10 μL) was added into the dispersion. Secondly, 10 μL of the catalyst ink was dropped onto the surface of GC and dried at room temperature for electrochemical test. 20 wt% commercial Pt/C was loaded on the electrode under the same procedure.

## Electronic supplementary material

Table S1, Table S2, Figure S1.
