# Supplementary material for: Three-Dimensional Multi-Doped Porous Carbon/Graphene Derived from Sewage Sludge with Template-Assisted Fe-pillared Montmorillonite for Enhanced Oxygen Reduction Reaction
Source: Sci Rep. 2017 Jun 23;7:4158. doi: 10.1038/s41598-017-03845-z (PMC5482810; doi:10.1038/s41598-017-03845-z)
Supplement: Supplementary file 1 — Table S1, Table S2, Figure S1. [file 41598_2017_3845_MOESM1_ESM.pdf]

Supplementary Information

for

Three-Dimensional Multi-Doped Porous Carbon/Graphene Derived from Sewage Sludge with Template-Assisted Fe-pillared Montmorillonite for Enhanced Oxygen Reduction Reaction

Meiqing Chen [a, b], Pingxiao Wu [a, b, c, d, e*], Liya Chen [a, b], Shanshan Yang [a, b], Langfeng Yu [a, b],

Yuefei Ding [a], Nengwu Zhu [a, d], Zhenqing Shi [a, e], Zehua Liu [a]

[a] *College of Environment and Energy, South China University of Technology, Guangzhou 510006, P.R. China*

[b] *The Key Lab of Pollution Control and Ecosystem Restoration in Industry Clusters, Ministry of Education, Guangzhou 510006, P.R. China*

[c] *Guangdong Provincial Engineering and Technology Research Center for Environmental Risk Prevention and Emergency Disposal, South China University of Technology, Guangzhou Higher Education Mega Centre, Guangzhou 510006, P.R. China*

[d] *Guangdong Environmental Protection Key Laboratory of Solid Waste Treatment and Recycling, Guangzhou 510006, China*

[e] *Guangdong Engineering and Technology Research Center for Environmental Nanomaterials, Guangzhou 510006, China*

[*] Corresponding author.

E-mail address: pppxwu@scut.edu.cn

Tel.: +86-20-39380538;

fax: +86-20-39383725

samples	C	C=C	C-N/C-O	C-O-C
Fe-Mt-SS-C	89.05	66.00	26.70	7.30
Mt-SS-C	82.48	69.20	24.00	6.80
Fe-SS-C	81.34	66.70	26.40	6.90
SS-C	83.26	67.10	19.30	13.60

Table S1. The percentages of total C, C=C, C-N/C-O and C-O-C calculated from XPS.

samples	N/%	pyridinic N/%	pyrrolic N/%	graphitic N/%	pyridinic N/%
Fe-Mt-SS-C	3.29	22.20	1.71	44.30	31.70
Mt-SS-C	4.56	16.30	27.90	39.30	16.40
Fe-SS-C	5.23	20.10	12.20	37.00	30.70
SS-C	4.81	12.20	24.70	30.40	32.70

Table S2. The percentages of total N, pyridinic N, pyrrolic N, graphitic N and pyridinic oxide (pyridinic N^{+}-O^{-}) calculated from XPS.

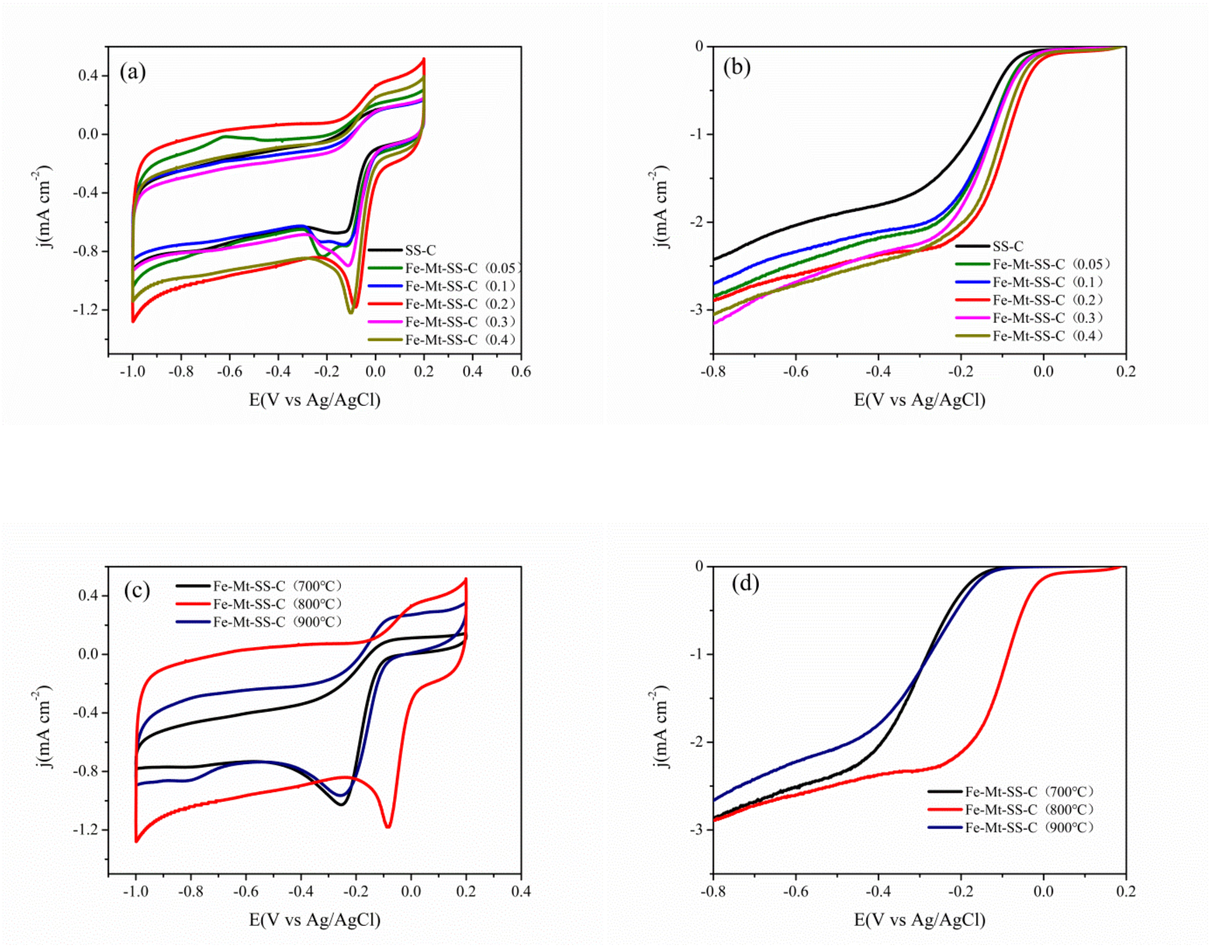

Figure S1. (**a**) Cyclic voltammograms and (**b**) RRDE voltammograms of as-obtained materials at different additive of Fe-Mt. (**c**) Cyclic voltammograms and (**d**) RRDE voltammograms of materials prepared under different temperature.
